# Dysfunctional Incidental Olfaction in Mild Cognitive Impairment (MCI): An Electroencephalography (EEG) Study

**DOI:** 10.3390/brainsci1010003

**Published:** 2011-10-28

**Authors:** Peter Walla, Cornelia Duregger, Lüder Deecke, Peter Dal-Bianco

**Affiliations:** 1 School of Psychology, Faculty of Science and Information Technology, University of Newcastle,Callaghan 2308 NSW, Australia; E-Mail: cornelia.duregger@newcastle.edu.au; 2 Department of Clinical Neurology, Medical University Vienna, Währinger Gürtel 18-20, 1090 Vienna, Austria; E-Mails: lueder.deecke@meduniwien.ac.at (L.D.); peter.dal-bianco@meduniwien.ac.at (P.D.–B.); 3 Faculty of Psychology, Biological Psychology Unit, University of Vienna, Liebiggasse 5, 1010 Vienna, Austria; 4 Applied Neuroscience, Neuroconsult e.U., Güntergasse 3/3, 1090 Vienna, Austria

**Keywords:** incidental olfaction, Mild Cognitive Impairment (MCI), electroencephalography (EEG)

## Abstract

Our study provides evidence that Mild Cognitive Impairment (MCI) is associated with olfactory dysfunction on both conscious and non-conscious levels. MCI patients and age-matched controls underwent a face processing task during which sympathy decisions had to be made via button presses. Incidentally, some of the faces were associated with a simultaneously presented odour. Although attention was paid to faces, brain activities were analysed with respect to odour *versus* no-odour conditions. Behavioural differences were found related to overall face recognition performance, but these were not statistically significant. However, odour-related neurophysiology differed between both groups. Normal controls demonstrated brain activity differences between odour and no-odour conditions that resemble difference activity patterns in healthy young participants as described in a previous magnetoencephalography (MEG) study [1]. They showed odour-related activity patterns between about 160 ms and 320 ms after stimulus onset and between about 640 ms and 720 ms. On the other hand, the patient group did not show any such difference activities. Based on previous research we interpret the early odour-related brain activity pattern in controls as being associated with subliminal olfaction and the later activity pattern with conscious olfaction. None of these were found in MCI patients, although it has to be emphasised that our sample size was rather small. We confirm previous findings about olfactory related dysfunction in patients with MCI and conclude from our findings that even subliminal odour-related information processing is impaired.

## 1. Introduction

Mild Cognitive Impairment (MCI) has been in discussion as a potential preclinical stage of Alzheimer's disease (AD) [2,3,4,5,6]. It is a quite frequent chronic condition in the elderly, but knowledge about factors that predict its development is limited. Prevalence estimates predict an increasing number of Alzheimer's disease in the near future due to the rapid growth of older age groups. This situation points out the need for preventive methods to at the best avoid the conversion from MCI to AD [7,8]. At the moment, effort is taken to find as many early markers as possible to support an early diagnosis and further to support an early treatment [9]. Recent studies showed the importance of multiple and continuous testing procedures in patients with MCI by combining early markers related to different aspects of anatomical change and physiological dysfunction [10].

To date, functional imaging studies demonstrate evidence for cognitive dysfunctions at early stages of AD and in MCI [11]. For example, when performing a cognitive task MCI patients have been found to show greater hippocampus activity while at the same time demonstrating a normal task performance when compared to healthy controls [12]. Püregger *et al.* [13] described magnetoencephalography (MEG) correlates in MCI patients in relation to non-semantic and semantic word encoding and following recognition performances. Although performances were similar between patients and controls they found neurophysiological signatures of functional compensation due to neural degeneration between about 250 ms and 450 ms after word onset.

Besides cognition-related early markers, one further function found to be affected is olfaction. Previous studies showed an early occurrence of deficits in various olfaction-related functions such as detection threshold and odour identification in MCI and in AD before clinical symptoms are fully developed [14,15,16,17,18]. These studies confirm the reliable contribution of olfaction-related investigations to broaden the spectrum of an early diagnosis.

Even neurophysiological investigations revealed reliable deviations from normal brain activity patterns [19]. Most of olfaction-related research included intentional conscious odour information processing. At the same time, research around the function of implicit memory [20] shows that even non-conscious information processing can be described by means of electroencephalography (EEG). In order to introduce an additional physiological indicator for an early diagnosis including non-conscious olfaction we intended to investigate rather incidental olfactory information processing in patients diagnosed with MCI. For this purpose we investigated dynamic odour-related information processing during simultaneous face-related information processing. This idea is based on the findings of previous magnetoencephalography (MEG) studies in healthy individuals [21,22,23,24]. For example, in healthy individuals it was shown that significant brain magnetic field differences occurred between faces with simultaneous odour stimulation and faces without simultaneous odour stimulation. These differences were found between about 200 ms and 300 ms after stimulus onset and between about 600 ms and 900 ms. The early brain magnetic field difference was due to a stronger magnetic field related to faces without odour than faces with a simultaneously associated odour [21]. On the other hand, between about 600 ms and 900 ms after stimulus onset face presentations with an associated odour evoked stronger brain magnetic fields than face presentations without an associated odour. The authors concluded that the early difference field reflects subliminal olfactory information processing whereas the later difference field reflects conscious olfaction. This conclusion is based on another previous study about conscious and non-conscious olfaction [25]. In that study, neurophysiological correlates of olfactory information processing were compared between two groups of study participants. Some of them reported conscious olfactory perception while others reported no conscious olfactory experiences during the course of the experiment. Strikingly, the late olfactory-related brain activity only occurred in the conscious perception group whereas the early olfactory-related brain activity was evident in both groups. For the present study we were motivated to use the above mentioned experimental paradigm including face and odour presentations to test whether or not MCI patients show the same pattern of results compared to normal controls by using the electroencephalography (EEG).

## 2. Methods

### 2.1. Participants

Eight patients suffering from MCI (mean age = 67.5 years) were included in this study. They were all defined by the following characteristics: Subjective memory impairment confirmed by an informant; objective memory impairment when compared with persons of similar age and education; normal general cognitive function; and normal activities of daily living [2]. They were all tested via the Crook test battery [26] twice (within 2 years), including the Mini-Mental State Examination (MMSE) [27]. MCI patients had a mean MMSE score of 28.78 (SD = 1.6). They were diagnosed as MCI if their Crook test performance was in at least one subtest more than 1½ standard deviations below the norm (same criteria as in [13]).

Eight age matched controls (mean age = 66.4 years) tested with the Crook test battery as well as with the MMSE were also invited to participate (mean MMSE score: 28.53 (SD = 1.2). No significant age difference occurred (*p* = 0.817) as well as no significant difference in MMSE score (*p* = 0.927). Controls had to have normal memory for their age and no psychiatric and no neuropathology history to qualify for participation.

All participants were right handed as shown by the results of Edinburgh Inventory [28]. They all gave their written informed consent for voluntary participation.

### 2.2. Procedure

Gray scale pictures of adult faces were visually presented on a computer monitor for 500 ms each with an inter stimulus interval of 5 s. During a first encoding phase the instruction was to classify each face as likeable or not likeable via button press. During this phase, 16 out of 74 presented faces were simultaneously associated with phenylethyl alcohol (rose-like smell), taking into account the ideal temporal distance between odour presentations to avoid olfactory habituation. For odour delivery a computer controlled olfactometer (Heinrich Burghart Elektro- und Feinmechanik GmbH, Wedel, Germany) was used (for a more detailed description see [1]). It embeds pulses of the odour into a constantly flowing air stream of controlled humidity and temperature. To ensure constant odour delivery via a small Teflon tube in the right nostril participants were instructed to breath through their mouth (velopharyngeal closure). A constantly flowing air stream including neutral room air was replaced by an air stream containing the odorant simultaneous to visual face presentation for the odour condition. For the control condition the replacing air stream had no odour in it. Via headphones acoustic white noise (80 Db) was presented to ensure olfactometer-related switching noises were not disturbing the procedure. After a short break of 2–3 min a face recognition test phase followed. All faces from the previous encoding phase were presented again together with the same number of new faces. No odour stimulation occurred. During this test phase the instruction was to decide for each face wether it was on the previous list or not. Presentation duration was again 500 ms and the inter stimulus interval was 3.5 s. Responses were given by button press.

In total, 4 such encoding and recognition blocks were provided for each participant to ensure a sufficient amount of data.

### 2.3. Data Acquisition

EEG data were recorded with a Neuroscan EEG system using 28 equally distributed Ag-AgCl scalp electrodes which were attached according to the international 10/20 system. Linked mastoids were used as reference. The sampling rate was 250/s and online filter settings were from DC to 100 Hz. Offline, a band pass filter from 0.3 Hz to 30 Hz was applied. Eye blinks were recorded via electrooculography (EOG) to allow for later eye blink-related artefact rejection. Single trials were defined as time windows with a constant length of 2.5 s. A period of 300 ms before face onset was used for baseline correction. All trials were averaged according to the following two conditions: (a) Face presentation during the encoding phase with simultaneous odour presentation; and (b) Face presentation during the encoding phase without simultaneous odour presentation. To ensure that both averages resulted from the same number of trials only the second last presentation before each face with simultaneous odour presentation was included into the data set instead of including all trials without simultaneous odour presentation. The same procedure was applied to MCI patients as well as normal controls.

### 2.4. Statistical Analysis

#### 2.4.1. Behavioural Data

To test whether both participant groups followed the given instruction we calculated overall face recognition performance. For this purpose, the mean number of falsely classified new faces (false alarms) was subtracted from the mean number of correctly recognised repeated faces (hits). A one-way ANOVA was calculated to test possible differences between patients and controls.

#### 2.4.2. EEG Data

From 0 s to 0.8 s after stimulus onset mean amplitude values were calculated for consecutive 40 ms time intervals. These mean values were used for statistical analysis. They were introduced to a repeated measures design ANOVA including the factor *condition* with two levels (face with odour, face without odour) and *electrode* with 28 levels. Participant group was introduced as between subject factor. Post-hoc, oneway ANOVA was used to highlight group differences for both conditions separately. All physiological data were also normalized according to McCarthy and Wood [29]. Normalised data were calculated with the same repeated measures and oneway ANOVA design. Thereby, the calculation of raw data is suggested to reflect possible quantitative amplitude differences between the conditions whereas the calculation of normalised data is suggested to reflect possible qualitative effects due to functional differences between conditions. T-maps (*t*-tests for every electrode location comparing both conditions) were created to demonstrate the distribution of physiological differences and Low-Resolution Brain Electromagnetic Tomography (LORETA; see [30]) was used to visualise related brain activities for time windows showing significant effects.

## 3. Results

### 3.1. Behavioural Data

**Figure 1:**
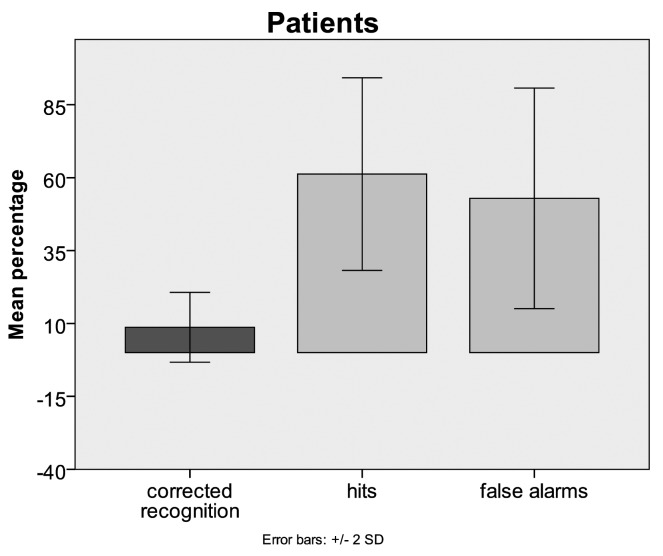

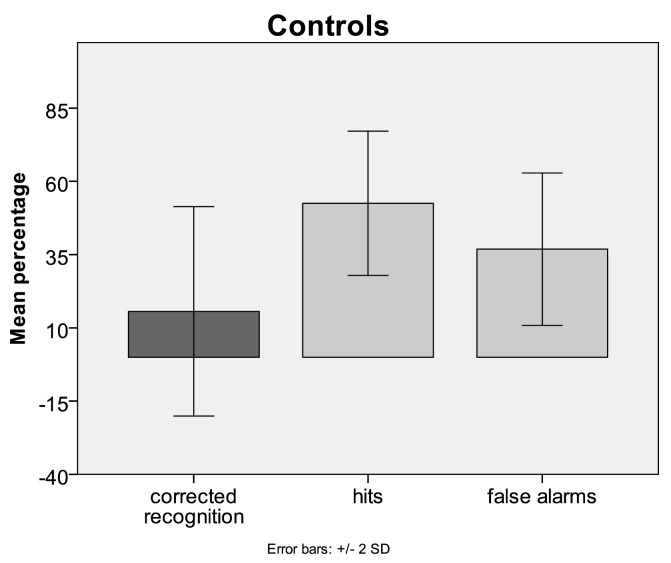
Relative overall face recognition performances for Mild Cognitive Impairment (MCI) patients and age-matched controls (corrected for guessing) (bars in dark grey). Only the number of false alarms shows a trend towards significant difference between MCI patients and controls. This might reflect a difference in response criterion. Patients are more liberal than controls in their decisions.

The mean percentage of false alarms in patients was 52.89 (SD = 18.9) and in controls it was 36.86 (SD = 13). On the other hand, the mean percentage of hits in patients was 61.24 (SD = 16.5) whereas it was 52.5 (SD = 12.3) in controls. Mean recognition performance corrected for guessing in patients was 8.68 (SD = 5.9) and for controls it was 15.63 (SD = 17.8) (Figure 1). The results of the oneway-ANOVA showed no significant differences between patients and controls. Although the actual mean recognition performance in controls was almost double as high as in patients, this difference did not turn out to be significant. This could be due to the relatively small sample size or due to inter individual variability. A slight trend towards a significant difference between patients and controls occurred for the mean percentage of false alarms (F = 3.902; *p*= 0.068) which was higher in patients than in controls.

### 3.2. EEG Data

Analysis of raw data revealed significant condition main effect and group interactions for the intervals 160 ms to 200 ms (*F* = 4.155; *p* = 0.049; η^2^ = 0.101; Greenhouse-Geisser corrected), 240 ms to 280 ms (*F* = 6.295; *p* = 0.017; η^2^ = 0.145; Greenhouse-Geisser corrected) and 280 ms to 320 ms (*F* = 4.803; *p* = 0.035; η^2^ = 0.115; Greenhouse-Geisser corrected). Analysis of the normalised data set revealed a significant condition*electrode interaction difference between patients and controls for the interval 680 ms to 720 ms (*F* = 2.405; *p* = 0.005; η^2^ = 0.061; Greenhouse-Geisser corrected).

Post-hoc, for the above mentioned intervals demonstrating significant condition main effects with group interaction we found no condition main effects in the patient group alone (not for raw and not for normalised data). On the other hand, in the raw data set of controls we found several significant condition main effects. They occurred for the intervals 160 ms to 200 ms (*F* = 7.345; *p* = 0.016; η^2^ = 0.329; Greenhouse-Geisser corrected), 200 ms to 240 ms (*F* = 5.117; *p* = 0.039; η^2^ = 0.254; Grenhouse-Geisser corrected), 240 ms to 280 ms (*F* = 6.516; *p* = 0.022; η^2^ = 0.303; Greenhouse-Geisser corrected), 280 ms to 320 ms (*F* = 7.802; *p* = 0.014; η^2^ = 0.342; Greenhouse-Geisser corrected) and 640 ms to 680 ms (*F* = 4.579; *p* = 0.049; η^2^ = 0.234; Greenhouse-Geisser corrected). One interval showed a significant condition*electrode interaction (680 ms to 720 ms; *F* = 2.582; *p* = 0.026; η^2^ = 0.147; Greenhouse-Geisser correction). Analysis of the normalised data set in controls revealed significant condition*electrode interactions for 4 time intervals (600 ms to 640 ms; *F* = 2.316; *p* = 0.029; η^2^ = 0.134; Greenhouse-Geisser corrected); 640 ms to 680 ms (*F* = 3.157; *p*= 0.004; η^2^ = 0.174; Greenhouse-Geisser corrected); 680 ms to 720 ms (*F* = 3.780; *p*< 0.001; η^2^ = 0.201; Greenhouse-Geisser corrected); 760 ms to 800 ms (*F* = 2.493; *p* = .017; η^2^ = 0.142; Greenhouse-Geisser corrected).

This clearly shows that the odour condition differs from the no-odour condition in controls, but not in patients.

**Figure 2:**
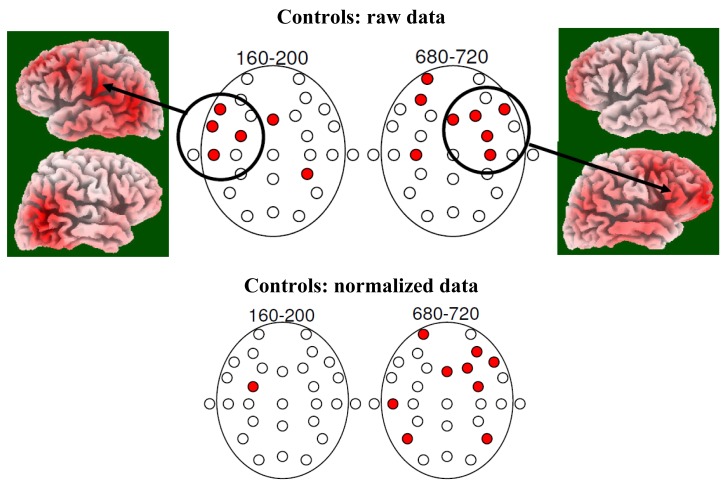
T-maps showing distributions of significant differences between the with-odour and the without-odour condition in age-matched controls. Note that controls show a number of significant effects at the left fronto-temporal area for the early time window and at the right fronto-temporal area for the later time window (raw data and normalised data). This situation matches nicely previous findings in young healthy study participants (Walla *et al.* 2003 [1]). In patients no such differences were found.

Figure 2 shows t-map results demonstrating the distribution of neurophysiological differences between the conditions faces without odour and faces with odour for controls (no such differences were found in patients) for both raw and normalised data. In addition, LORETA solutions are shown demonstrating localised brain activities related to the condition faces with odour in controls. It can be seen that between about 160 ms and 200 ms after stimulus onset significant difference activity as shown in the respective t-map is over left fronto-temporal electrode locations. Later, between about 680 ms and 720 ms after stimulus onset the main brain activity difference distribution is focussed at the right frontal area.

## 4. Discussion

In the meantime, a critical number of solid empirical evidence demonstrates olfactory impairment in MCI patients (e.g., [31,32,33]). As mentioned in the introduction, any further evidence is highly appreciated as well as any better understanding of it. The present study provides further neurophysiological evidence of olfactory dysfunctions in patients with MCI and highlights that dysfunctions are evident for both conscious and unconscious olfactory information processing. According to a recent review the motivation to use olfactory dysfunction as an index for the AD should be strongly supported [34]. Already 13 years ago a meta-analysis of more than 40 studies revealed that the AD is indeed associated with significant olfaction-related dysfunctions [35]. Since MCI patients have a higher risk of developing AD than aged matched controls it is suggested that olfactory dysfunction should be used as a biomarker in this neurodegenerative impairment as well.

### 4.1. Behaviour

The absolute face recognition performances differ qualitatively, but according to our statistical analysis they are similar between MCI patients and controls. Due to the amnestic-type nature of our patients one might expect significant face recognition performance differences. Anyway, recognition performance was not important to us. All we wanted to know was whether both groups followed the instructions given to them, which they obviously did. Since both groups demonstrated above chance detection of repeated face presentations we can confirm that. However, it might be useful to mention that in a previous study [13] similar recognition performances between MCI patients and controls were found for shallow (non-semantic) and for deep (semantic) encoded words. It therefore seems that regardless of what information is to be encoded MCI patients will subsequently recognise it just about as accurate as normal controls. This might be expected due to the mildness of cognitive decline related to MCI, despite the fact that diagnose-specific memory tests actually confirm some sort of memory impairment (amnestic-type).

The present study provides evidence for differences between MCI patients and controls with respect to another behavioural measure, namely response criterion. Recognition performance is calculated as correctly recognised repetitions (hits) minus false alarms (incorrectly judged new items). The result of this substraction is recognition performance corrected for guessing. As a matter of fact, different ratios of hits and false alarms can lead to equal corrected recognition performances. Such different ratios reflect different response criteria. In the present study, MCI patients had a higher rate for correctly recognised repeated faces (hits) than controls. However, their false alarm rate was higher too and finally calculated recognition performances were not statistically different between the two groups. The higher hit and false alarm rates in MCI patients reflect a more liberal response criterion compared to controls. This means that the patients were more biased towards judging a presented face as repeated than controls. At this stage it remains unclear how this finding can be translated into a clinically relevant theory, but we believe that future studies focussing on this behavioural phenomenon will provide some insight.

Finally, comparing the present results with previous findings a linear decline in face recognition performance after deep encoding can be seen from healthy young participants (above 20%; [1]) over older participants (about 15%) to MCI patients (about 8%) as found in the present study.

### 4.2. Electrophysiology

**Figure 3:**
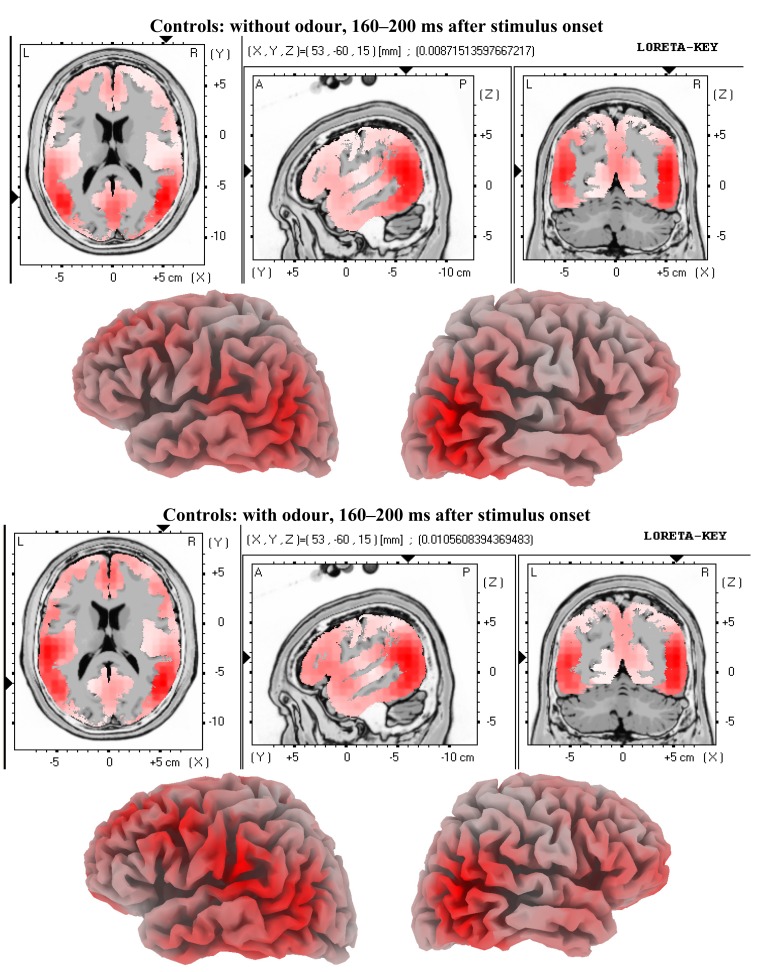
LORETA solutions for the early odour-related effect in controls. Maximum brain activity difference between odour *versus* no-odour can be seen at the left fronto-temporal area.

**Figure 4:**
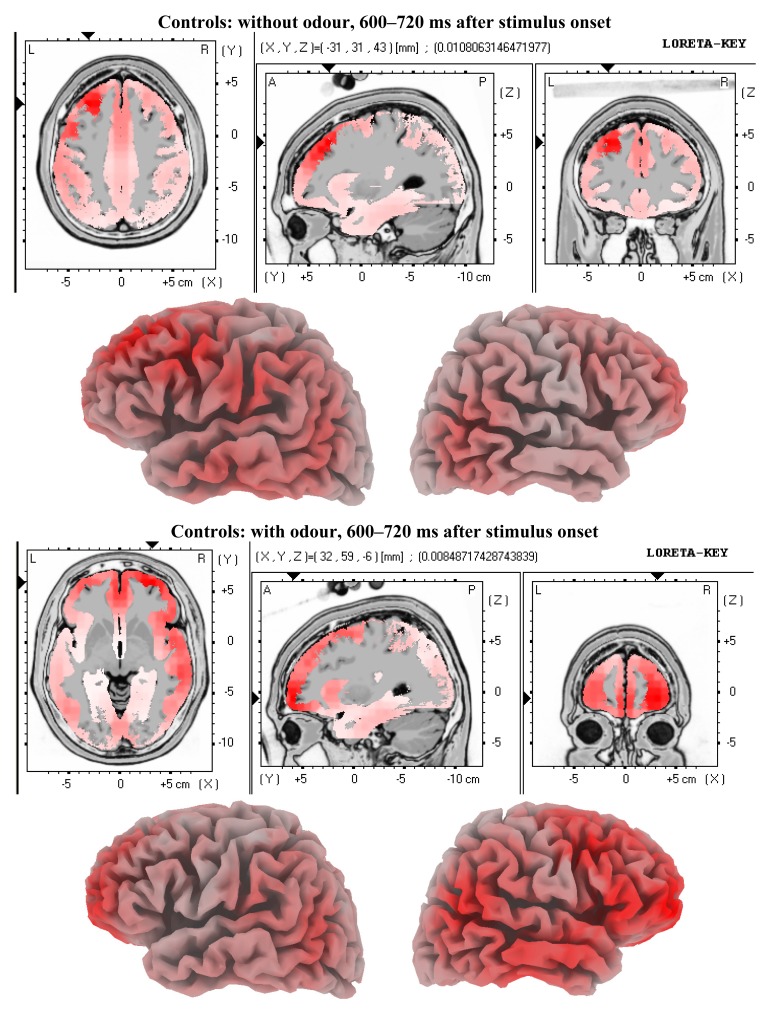
LORETA solutions for the late odour-related effect in controls. Maximum brain activity difference between odour *versus* no-odour can be seen at the right frontal area.

We tested the hypothesis that MCI patients demonstrate different brain activity patterns related to incidental olfactory information processing compared to age-matched controls due to their known olfactory impairment. This can be confirmed. In comparison to previous findings [1] the present study too revealed significant brain activity differences between the conditions faces without odour and faces with odour in healthy age-matched controls. The pattern of brain activity differences in our control group looks convincingly similar to those in young healthy adults as demonstrated by Walla *et al.* [1]. Figures 3 and 4show visualised brain activities of both conditions (with odour and without odour) in the control group for both time windows for which significant effects were found. However, these apparently normal difference brain activity patterns are missing in MCI patients. At this point is has to be emphasised again that this finding is based on a relatively small sample size. However, our selection criteria with respect to patients were very rigorous and conservative and thus supporting significance of possible differences compared to normal controls.

Given that the sense of olfaction has been shown to be impaired in MCI patients [15,16,14,18,34,35] our finding might not be surprising, but two details are important to note. The first detail relates to the known distinction between conscious and subconscious olfactory information processing as suggested by Walla *et al.* [25]. Our study provides evidence that both conscious and subconscious olfaction seem to be impaired in MCI patients. This is due to the fact that neither early nor late odour-related effects occurred in our patient group. In controls, we found an early and a late odour-related effect. The early effect occurred between about 160 ms and 300 ms after stimulus onset and the late effect occurred between about 640 ms and 800 ms after stimulus onset. Both time windows nicely match the two chronological effects found in young healthy study participants [1,25]. We can therefore complete previous reports about olfactory dysfunction in MCI patients by amending that not only conscious aspects of olfaction are impaired, but also subconscious aspects of it. The second detail refers to the fact that no odour-related task had to be performed by our study participants. Instead, our participant's attention was focussed on face processing while all odour-related information processing was purely incidental. Although we cannot provide any proof that being aware of olfactory testing has an effect on olfactory performance and thus might manipulate test results we can still argue that just in case our approach does provide an objective alternative to investigate odour-related processing in the human brain.

While our study adds evidence about olfactory dysfunction in MCI patients, it does not yet help to discriminate between MCI patients who develop AD and those who do not. For this purpose a long term follow-up study is needed. In general, it seems promising to use olfactory dysfunction as an early marker for MCI and AD as well as for an indicator for conversion from MCI to AD as shown by Devanand *et al.* [10,36]. They conclude that in patients with MCI, olfactory identification deficits, particularly with lack of awareness of olfactory deficits, may have clinical utility as an early diagnostic marker for Alzheimer's disease.
